# 90. Impact of anti-CD20 therapy on COVID-19 in immunocompromised patients: A nationwide cohort study in Korea

**DOI:** 10.1093/ofid/ofae631.027

**Published:** 2025-01-29

**Authors:** Chan Mi Lee, Suhyun Kim, Seungyeon Kim, Jiyeon Bae, Hyeon Jae Jo, Chang Kyung Kang, Pyoeng Gyun Choe, Wan Beom Park, Nam Joong Kim

**Affiliations:** Seoul National University College of Medicine, Seoul, Seoul-t'ukpyolsi, Republic of Korea; Seoul National University Hospital, Jongno-gu, Seoul-t'ukpyolsi, Republic of Korea; Dankook University, Cheonan, Ch'ungch'ong-bukto, Republic of Korea; Ewha Womans University College of Medicine, Seoul, Seoul-t'ukpyolsi, Republic of Korea; Seoul National University College of Medicine, Seoul, Seoul-t'ukpyolsi, Republic of Korea; Seoul National University College of Medicine, Seoul, Seoul-t'ukpyolsi, Republic of Korea; Seoul National University College of Medicine, Seoul, Seoul-t'ukpyolsi, Republic of Korea; Seoul National University College of Medicine, Seoul, Seoul-t'ukpyolsi, Republic of Korea; Seoul National University College of Medicine, Seoul, Seoul-t'ukpyolsi, Republic of Korea

## Abstract

**Background:**

During the coronavirus disease 2019 (COVID-19) pandemic, we observed that patients received anti-CD20 therapy progressed to severe pneumonia and underwent persistent viral shedding. We aimed to identify the association between anti-CD20 therapy and severe acute respiratory syndrome coronavirus 2 (SARS-CoV-2) infection, as well as the outcomes of COVID-19.

**Figure:**
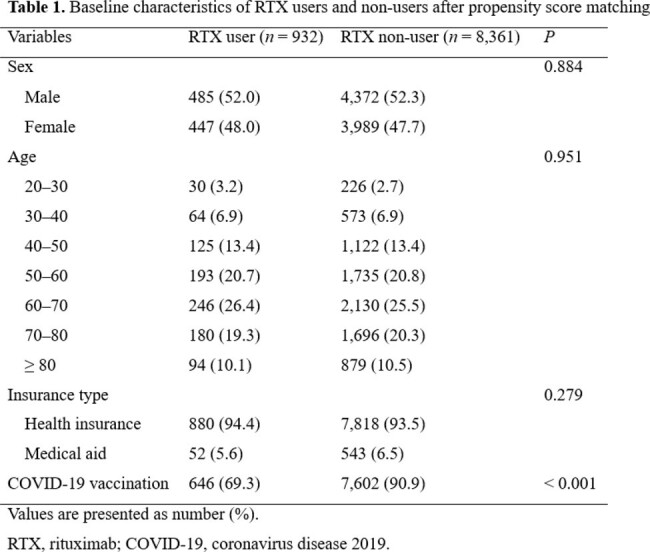

**Methods:**

Using claims data derived from the Korean National Health Insurance Service, we conducted a matched nationwide retrospective cohort study. We included individuals aged ≥ 20 years diagnosed with conditions for which anti-CD20 therapy could be considered from January 2020 to December 2021. We defined the exposure variable by prescribing rituximab (RTX) at least once according to the database. COVID-19-related outcomes included hospitalization for COVID-19, ICU admission, mechanical ventilation administration, and all-case death. Patients prescribed RTX and those not prescribed RTX were defined as RTX users and non-users, respectively. The RTX users and non-users were 1:10 propensity score matched using sex, age, insurance type, and comorbidities. Logistic regression analyses were used to estimate the association between RTX and SARS-CoV-2 infection, as well as COVID-19-related outcomes.

**Figure:**
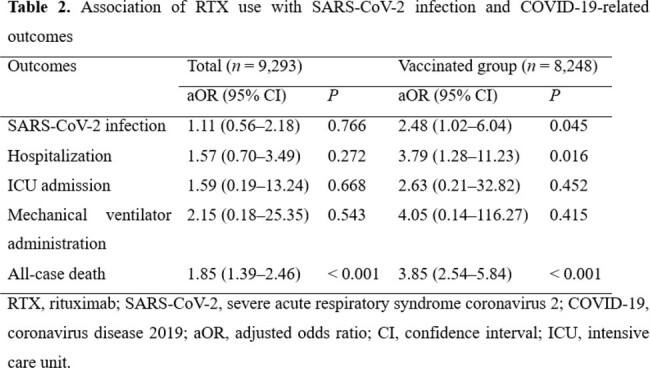

**Results:**

After 1:10 propensity score matching, 932 RTX users and 8,361 RTX non-users were analyzed. The proportion of vaccinated individuals was significantly lower in RTX users than in RTX non-users (69.3% vs. 90.9%; *P* < 0.001). RTX use was associated with an increased risk of all-case death (adjusted odds ratio [aOR] 1.85, 95% confidence interval [CI] 1.39–2.46, *P* < 0.001). In the subgroup analysis for vaccinated individuals, the risk of SARS-CoV-2 infection was significantly greater for RTX users compared to non-users (aOR 2.48, 95% CI 1.02–6.04, *P* = 0.045), and RTX use was also associated with a significantly increased risk of hospitalization (aOR 3.79, 95% CI 1.28–11.23, *P* = 0.016) and all-case death (aOR 3.85, 95% CI 2.54–5.84, *P* < 0.001).

**Conclusion:**

The use of RTX was associated with an increased risk of all-cause death in immunocompromised patients. Among vaccinated individuals, RTX users were at an increased risk of SARS-CoV-2 infection, as well as an increased risk for hospitalization of COVID-19 and all-cause death.

**Disclosures:**

**All Authors**: No reported disclosures

